# A Sustainable Solution to Obtain P-K-Mn Glass Fertilizers from Cheap and Readily Available Wastes

**DOI:** 10.3390/ijerph18126585

**Published:** 2021-06-18

**Authors:** Cosmin Vancea, Giannin Mosoarca, Simona Popa

**Affiliations:** Faculty of Industrial Chemistry and Environmental Engineering, Politehnica University Timisoara, Bd. V. Parvan No. 6, 300223 Timisoara, Romania; cosmin.vancea@upt.ro

**Keywords:** glass fertilizers, cheap wastes, wood ash, manganese sludge, plant growth parameters

## Abstract

The sustainable economy framework imposes the adoption of new ways for waste reuse and recycling. In this context, this paper proposes a new alternative to obtain glass fertilizers (agriglasses) by reusing two cheap and easily available wastes, wood ash and manganese rich sludge resulting from drinking water treatment processes for groundwater sources. Glasses were obtained using different amounts of wastes together with (NH_4_)_2_HPO_4_ and K_2_CO_3_ as raw materials. The P-K-Mn nutrient solubilization from the obtained glasses was investigated using a citric acid solution. The kinetics of the leaching process was studied after 1, 7, 14, 21 and 28 days, respectively. The intraparticle diffusion model was used to interpret kinetic data. Two distinct stages of the ion leaching process were recorded for all of the studied compositions: first through intraparticle diffusion (the rate-controlling stage) and second through diffusion through the particle–medium interface. The fertilization effect of the obtained agriglasses was studied on a barley crop. The specific plant growth parameters of germination percentage, average plant height, biomass and relative growth rate were determinate. The positive impact of the agriglasses upon the plants biomass and relative growth rate was highlighted. The effects of agriglasses can be tuned through glass compositions that affect the solubility of the nutrients.

## 1. Introduction

Each year, human activities generate large amounts of wastes that have a negative impact on the environment and must be stored or treated [1]. Their minimization can be achieved by reusing and recycling, which will lead to the reduction of dependence on natural resources and the achievement of sustainable development objectives [2,3]. In this context, the circular economy concept emphasizes the use of renewable energy resources and also the reuse of wastes in production cycles leading to an important decrease in waste and economic advantages [4,5,6,7]. If wastes conversion produces new and marketable products, a financial benefit is added to the ecological benefits.

Wood is a natural resource which has been widely used in various fields since the Paleolithic period. Large amounts are burned, in various industrial processes or in households, generating around 10% ash. Storage of this waste generates environmental problems and significant costs, respectively. Therefore, the reuse of ash has gained an increased interest. Depending on the composition and geographical region, ash has been used in agriculture (soil amendment), as forest soil amendment, to control the odor and pH of some wastes, to remove pollutants from water and to produce ceramics and building materials (cement base and road base material) [8,9,10,11,12,13,14,15,16,17,18,19,20,21,22,23,24,25].

Drinking water treatment processes for groundwater sources rich in iron and manganese have an acceptable hardness and generally include oxidation and removal of iron and manganese precipitates formed by two-stage filtration (in the first stage iron is retained and in the second stage the manganese) [26,27,28]. The filters that wash waters from the second stage contain large amounts of manganese. After decantation, a rich manganese dioxide sludge is obtained.

The objective of this research was to obtain agriglasses, a useful and marketable product, using two wastes: wood ash and manganese rich sludge. The glass fertilizers (agriglasses) are an eco-friendly alternative to classic chemical fertilizers, with the advantages of a high assimilation grade by plants, a slow and controlled nutrient release and the fact that they do not generate a residual soil and water pollution, which is the latest concept in fertilizer technology [29,30,31,32]. The controlled fertilizing effect is based on tailoring the chemical degradation depending on the glass chemical composition and granularity. The glass matrices incorporate both K, P, Mg, Ca macroelements and (B, Fe, Mo, Cu, Zn, Mn) microelements required for the growth of plants [29,30,31,32,33,34,35,36,37]. The agriglasses presented in scientific literature usually required raw materials and chemical reagents, affecting the marketable value of the obtained products. We propose a new approach for glass fertilizer synthesis, respecting one of the most important requirements for sustainable development and for circular economy, i.e., the reuse of anthropic generated wastes.

## 2. Materials and Methods

Wood ash from fir wood (*Abies pectinata*) combustion in a home fireplace was used. After a preliminary drying at 40–60 °C, wood ash was ground and passed through a 1 mm sieve. The wood ash main elements were determined after digestion with aqua regia by inductively coupled plasma mass spectrometry (ICP-MS) using a Perkin Elmer NexION 350× spectrometer. Phosphorous concentration was determined with the vanadomolybdophosphoric acid method using UV–visible spectrophotometer Specord 200 Plus. The calculated oxide composition is presented in Table 1.

The second waste used as precursor for agriglass synthesis was the sludge resulting from the settling of the filter washing waters from the manganese removal stage, collected from the Timisoara drinking water treatment plant for groundwater with high content of iron and manganese. After calcination at 750 ° C for 6 h, the waste composition was determined using an X-ray fluorescence Niton XL 3 analyzer as follows: 90.93% Mn^2+^ and 9.07% Fe^3+^.

A composition of necessary phosphorous–potassium glasses, both essential macronutrients for plants, was added using (NH_4_)_2_HPO_4_ and K_2_CO_3_ as raw materials of analytical grade. The wood ash amount used for the agriglass synthesis and the corresponding molar oxide composition of the obtained vitreous fertilizers are presented in Table 2.

The precursors were weighed, mixed together, loaded into porcelain crucibles and then melted at 1000 °C for 90 min using a Nabertherm HTC08/16 electric furnace. The melts were stirred to ensure good homogeneity and rapidly cooled by casting on brass plates. The obtained glasses were milled and sieved, and the fraction having a particle diameter under 0.25 mm was selected for this study.

The agriglasses’ chemical activity was determined by measuring their dissolution in 2 wt.% citric acid solution that simulates the behavior of organic compounds released by the plant roots and extracts the useful material from fertilizers [38]. The solid:liquid weight ratio was 1:100. The K, P and Mn concentrations, leached after 1, 7, 14, 21 and 28 days were determined using a using a Bruker Aurora M90 Inductively Coupled Plasma Mass Spectrometer. The pH was measured using a Mettler Toledo pH-meter.

The soil used in the tests was collected as the surface layer (0–15 cm), from a public garden in Timisoara town. The main soil characteristics are: pH_H2O_ = 6.8, sand = 35.3%, silt = 27.7% and clay = 37% [39].

Barley (*Hordeum vulgare* L) was purchased from a local farmer near Timisoara. After a previous sterilization using 80% (*v/v*) ethanol and rinsing with double distilled water, the seeds were completely dried at room temperature.

The effect of the synthesized glasses as fertilizers was tested using a pot experiment on barley. Parallel soil samples were seeded with the same number of seeds. Three grams of each agriglass composition (corresponding to a surface doze of 0.08 g cm^−2^) were applied on the soil surface. For a correct analysis of the results, a soil control sample without agriglass addition was prepared for comparison. For each glass composition, there were three independent replicates. All of the pots were periodically watered using the same volume of tap water. On the 28th day after sowing, the plants were harvested and the germination percentage, average plant height, biomass and relative growth rate were determinate.

The relative growth rate (RGR) was calculated with Equation (1):

RGR = (ln W_2_ − ln W_1_)/(t_2_ − t_1_) (1)

where W_1_ is fresh biomass of plants at time one (g), W_2_ is fresh biomass of plants at time two (g), t_1_ is time one (days) and t_2_ is time two (days) [40].

The calculated data are the mean of three independent replicates for each studied plant growth parameter. Before running the ANOVA analysis, equal variances tests (multiple comparisons and Levene’s methods) were performed, which indicated that the samples had equal variances. One-way analysis of variance (ANOVA) was used in order to assess significant differences in the glass fertilizers compositions. Considering *p* < 0.01 as a significant value, a comparison of mean using the least significant different test was calculated for *p*-values. Minitab 19 software was utilized to perform the required calculations.

## 3. Results and Discussion

### 3.1. UV–VIS Agriglass Characterization

**Figure 1:**
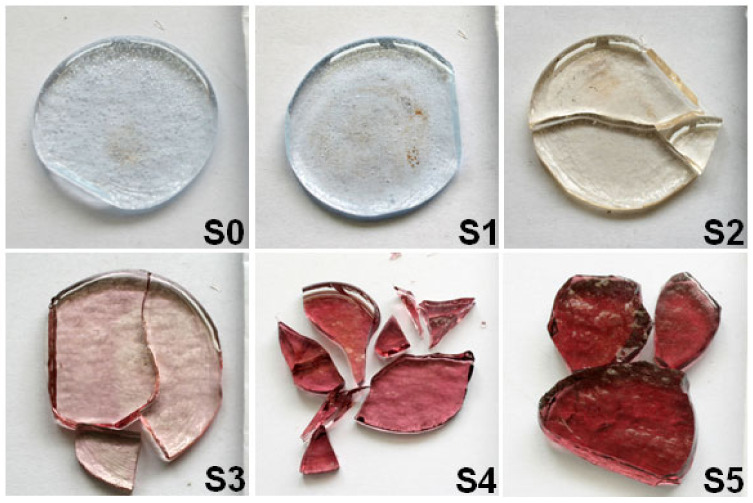
Synthesized agriglass samples.

**Figure 2:**
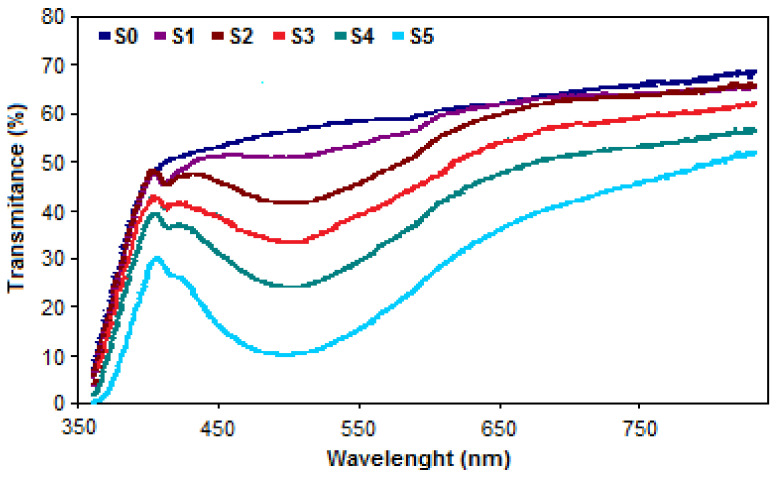
UV–VIS spectra of the obtained agriglass samples.

The obtained glass samples before milling are presented in Figure 1. The color changed from colorless for the first two samples to different shades of pink characteristic to the Mn^3+^ ion. The presence of this ion in the glass matrix is confirmed by the broad asymmetric band centered at about 480 nm [41,42] on the UV–VIS spectra presented in Figure 2. The double peak around 415 nm corresponds to the Mn^2+^ [43,44] ion that appears in glass according to the redox reaction: Mn^2+^ ↔ Mn^3+^ + e^−^.

### 3.2. Chemical Activity

The dissolution of phosphate glasses is a complex reaction-controlled process, based on the breakage of P-O-P bonds in the phosphate vitreous network within the hydrated layer and the extraction of ions from the glass matrix. The hydrolysis of phosphate groups can be described by the following reaction [33]:

(2) $$-\overset{\left|\right|}{\underset{|}{P}}-O-\overset{\left|\right|}{\underset{|}{P}}-+\;H-\mathrm{OH}\;\to -\overset{\left|\right|}{\underset{|}{P}}-\mathrm{OH}\;+\mathrm{HO}-\overset{\left|\right|}{\underset{|}{P}}-$$

Complex multicomponent glass leaching processes are based on the hydrolytic cleavage of various bonds in the glass network which have different hydration energies. The weakest metal ion–non-bridging oxygen bonds break first during the dissolution process [45].

(3) $$-\overset{\left|\right|}{\underset{|}{P}}-O-\overset{\left|\right|}{\underset{|}{P}}-\mathrm{Me}\;-\overset{\left|\right|}{\underset{|}{P}}-O-\overset{\left|\right|}{\underset{|}{P}}-+\;H-\mathrm{OH}\;\to -\overset{\left|\right|}{\underset{|}{P}}-\mathrm{OH}\;+\mathrm{HO}-\overset{\left|\right|}{\underset{|}{P}}-+{\mathrm{Me}(\mathrm{OH})}_{2}$$

The ion exchange rate through the hydration layer and the water penetration into the glass depends on the surface concentration of the interdiffusing ions, the multicomponent interdiffusion coefficient and the exchange potential of the interdiffusing species at the exchange site [34].

**Figure 3:**
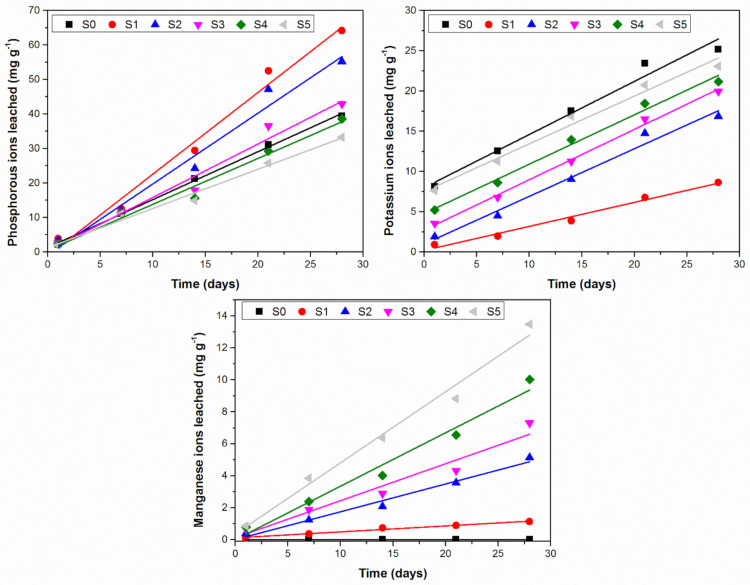
Ions dissolution from the studied glasses.

The kinetics curves for phosphorous, potassium and manganese ion dissolution from the synthesized glass fertilizers are presented in Figure 3.

All sets of curves for the three studied ions leached from the agriglasses at each considered term show a quasi-linear behavior, with the linear fitting parameters summarized in Table 3.

The intraparticle diffusion model was used to correlate the experimental data on phosphorus, potassium and iron ions leached from the obtained agriglasses in the 2% citric acid solution [46]. The diffusion equation is described by Equation (2) [47]:

q_t_ = k_t_ × t^1/2^ + C (4)

where k_t_ is the intraparticle diffusion rate constant (mg g^−1^ h^−1/2^) and C is the intercept, related to the thickness of the boundary layer. Higher C values indicate greater boundary layer effect [48].

The term q_t_, describing the leached ion amount per gram of glass, was calculated using Equation (3):

q_t_ = V(C_0_ − C_t_)/m (5)

where: C_0_ and C_t_ are initial concentration and the concentration corresponding to a considered time t (mg L^−1^), V is citric acid solution volume (L) and m is the mass of agriglass sample (g).

**Figure 4:**
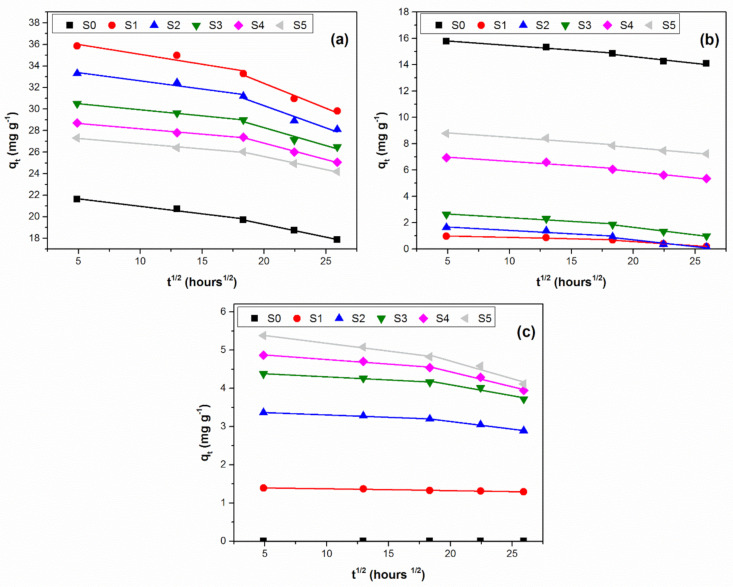
Intraparticle diffusion model plot for the investigated glasses: (**a**) phosphorous ions; (**b**) potassium ions; (**c**) manganese ions.

The plots q_t_ = f(t^1/2^) are illustrated in Figure 4 for phosphorous, potassium and manganese ions.

The plots q_t_ = f (t^1/2^) for the dissolution of three studied ions from all investigated glass fertilizers show a double linearity described by the specific fitting parameters presented in Table 4.

The very good linearity of the q_t_ = f(t^1/2^) plots is highlighted by the values of the coefficients of determination over 0.95, confirming the applicability of the intraparticle diffusion model to describe the ions dissolution. This behavior, previously mentioned in literature [49,50], indicates that two processes influence the ion leaching in the citric acid solution. The first corresponds to the diffusion through the vitreous matrix and the second to the diffusion through the glass particle–medium interface.

For all of the synthesized glasses, the linear regression slope for the first diffusion stage is significantly lower compared to that for the second step, indicating that the rate-controlling stage is intraparticle diffusion. All studied glasses show an intraparticle diffusion rate constant k_t_ lower in the first step compared to the second step due to the fact that intraparticle diffusion through the vitreous matrix is a slower process compared to diffusion at the glass–medium interface.

The thickness of the boundary layer is higher for the second ion dissolution step for all the glasses, indicating the influence of the interface leaching mechanism in this step.

### 3.3. pH Evolution

**Figure 5:**
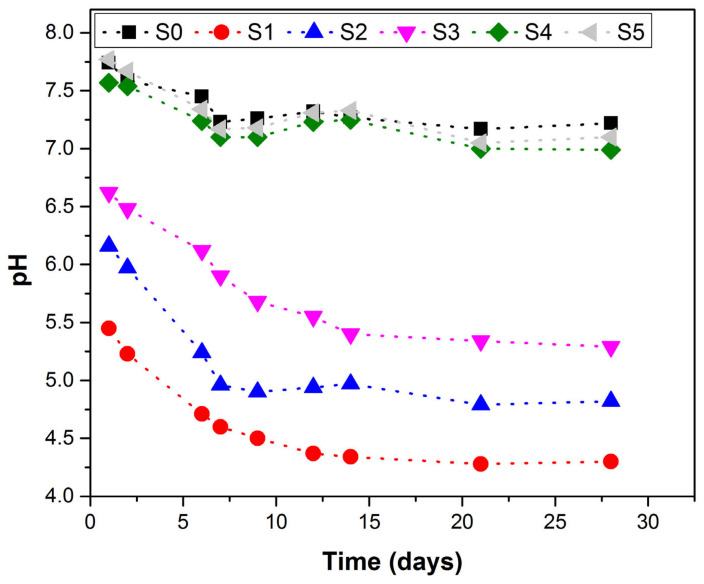
pH evolution over time for studied glasses.

The evolution of pH over time for all of the synthesized glass fertilizers using distilled water as solvent is presented in Figure 5.

A similar allure of the pH = f(time) curves can be observed for all the studied glasses: an initial pH drop recorded after seven days and a stabilization range for longer terms. The decrease in pH can be attributed to the massive phosphorous leaching compared to a lower amount of alkali released at short terms. The future network degradation, due to a higher dissolution rate based on the previously described mechanism, leads to a larger amount of alkali ions extracted from the vitreous matrix and a pH range.

Two distinct behaviors can be distinguished, the first one for glasses S1–S3 and the second one for the glasses S0, S4 and S5, based on their structure differences. The first group of glasses, which have an O/P ratio between 1.76 and2.48, belongs to ultraphosphate glasses (O/P ratio < 3), while the second group, characterized by an O/P ratio between 2.78 and 3.09, is close to metaphosphate glasses (O/P ratio = 3) [51]. The ultraphosphate glasses, much more susceptible to network degradation by hydrolysis, generate larger phosphorous ion amounts in the leaching medium, which leads to a significant decrease in pH. The samples from the second group tend to have a more stable structure, characteristic of metaphosphate glasses, and are less affected by hydrolysis. Less ions are leached from the vitreous matrix, which leads to a narrow variation of pH values over the considered time.

### 3.4. Effects on Plants Growth

The practical applicability of these agriglasses was studied using barley (*Hordeum vulgare* L.), a well-known bioindicator plant [52,53,54,55].

**Figure 6:**
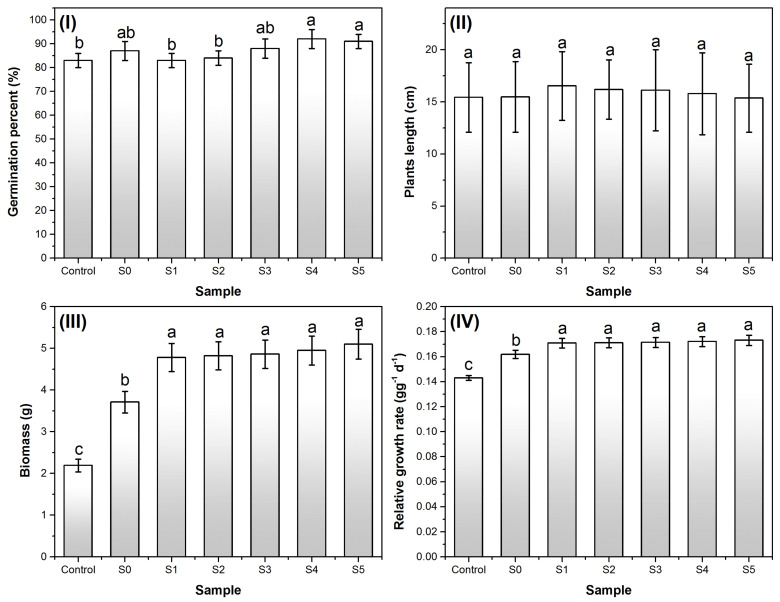
The main specific parameters of plants growth: (**I**) germination percent; (**II**) plant average length; (**III**) biomass; (**IV**) relative growth rate. Values are expressed as means of three replicates and error bars represent the standard deviation. Columns denoted by different letters indicated significant (*p* < 0.01) differences among different agriglass compositions.

Figure 6I illustrates the germination percentage of the plants for the control sample (without agriglass) and for the samples fertilized using the synthesized agriglasses. This parameter was higher by 1% to 10% in the samples with agriglass compared to the control sample. The average length of the harvested plants in the fertilized samples (Figure 6II) does not differ significantly from the control sample; instead, the biomass increases substantially, by 100% to 200% (Figure 6III). The relative growth rate (RGR) increases are also significant in the fertilized samples (Figure 6IV). The obtained results highlight the potential positive impact of the proposed agriglasses on agricultural crops.

The positive results obtained in the laboratory experiments represent the premise for testing these fertilizers in the field, in real crop conditions and also on other plant species.

## 4. Conclusions

In the context of sustainable development, this paper proposes a new way to reuse two cheap wastes available in large quantities, wood ash and manganese rich sludge from the drinking water treatment process, for the synthesis of glass fertilizers. Six different compositions were synthesized considering energy efficiency criteria in choosing the fusion thermal parameters, taking into account the energy-consuming character of the glass melting process. Kinetic studies of the solubilization of potassium, phosphorus and manganese ions, important in plant nutrition, were performed at five terms: 1, 7, 14, 21 and 28 days, respectively. The interpretation of kinetic data was made using the intraparticle diffusion model. All of the synthesized glasses present two distinct stages of the ion leaching process: first through intraparticle diffusion and second through diffusion through the particle boundary, the rate-controlling stage being the intraparticle diffusion. The main parameters for the intraparticle diffusion model for phosphorous, potassium and manganese ions leach were calculated. The importance of the vitreous matrix structure on the behavior of these fertilizers is emphasized by the pH evolution over time. The ultraphosphate glasses (O/P ratio < 3) are more susceptible to network degradation by hydrolysis, while the metaphosphate glasses (O/P ratio = 3), having a more stable structure, are less affected by hydrolysis. The practical applicability of the studied glasses as fertilizers was highlighted by the positive effect on the main specific plant growth parameters for a barley crop. Significant beneficial effects on the biomass and relative growth rate were recorded for the S1–S5 samples, containing manganese as a microelement. It can be concluded that valuable fertilizers with controlled solubility can be obtained from common wastes. The glass composition is the main factor that controls the leaching of the nutrients from the vitreous matrix.

## Figures and Tables

**Table 1:** Oxide composition of used fir wood ash.

Oxide	SiO_2_	Al_2_O_3_	Fe_2_O_3_	MnO_2_	Na_2_O	K_2_O	CaO	MgO	P_2_O_5_
(%)	38.52	2.27	1.74	2.67	0.27	15.57	26.63	7.40	4.93

**Table 2:** Wood ash content and corresponding oxide composition of the studied glass fertilizers.

Sample	S0	S1	S2	S3	S4	S5
Wood ash amount (mg g^−1^)	0	20	40	60	80	100
Corresponding agriglasses’ molar oxide composition						
SiO_2_ (%)	3.72	5.43	8.23	10.59	12.00	13.05
Al_2_O_3_ (%)	0.00	2.49	3.78	4.87	5.51	6.00
Fe_2_O_3_ (%)	0.00	0.13	0.19	0.24	0.28	0.30
MnO (%)	0.00	2.84	6.13	7.66	8.28	8.91
Na_2_O (%)	16.55	22.91	18.72	16.42	14.22	12.40
K_2_O (%)	18.99	1.53	2.32	3.63	8.81	10.97
CaO (%)	15.94	1.95	2.98	3.82	4.34	6.77
MgO (%)	13.39	2.26	11.36	12.81	12.37	11.68
P_2_O_5_ (%)	31.42	60.46	46.29	39.95	34.18	29.93

**Table 3:** Linear regression equations and corresponding coefficients of determination for ions leaching dissolution.

Sample	S0	S1	S2	S3	S4	S5
Ion Leached	Phosphorous					
Equation	y = 1.3888x + 1.2904	y = 2.3681x − 1.1212	y = 2.0519x − 0.7908	y = 1.5497x + 0.3149	y = 1.3305x + 0.5071	y = 1.1357x + 1.2584
R²	0.9982	0.9853	0.9765	0.9722	0.9802	0.9845
Ion leached	Potassium					
Equation	y = 0.6598x + 7.9997	y = 0.2985x + 0.1971	y = 0.5917x + 0.994	y = 0.5917x + 0.994	y = 0.6137x + 4.7697	y = 0.5928x + 7.5082
R²	0.978	0.9846	0.9827	0.9827	0.9896	0.9805
Ion leached	Manganese					
Equation		y = 0.0373x + 0.1163	y = 0.1738x + 0.0092	y = 0.231x + 0.1296	y = 0.3341x + 0.0054	y = 0.4444x + 0.3635
R²		0.9807	0.9826	0.9499	0.9758	0.9849

**Table 4:** Intraparticle diffusion parameters for both steps for phosphorous, potassium and manganese ions leaching kinetics.

Sample	S0	S1	S2	S3	S4	S5
Ion leached	Phosphorous					
	Step 1					
k_t_	0.1393	0.1837	0.1521	0.1125	0.0993	0.0971
C	22.361	36.916	34.140	31.057	29.159	27.754
R²	0.9758	0.9841	0.9611	0.9995	0.9924	0.9864
	Step 2					
k_t_	0.2391	0.4613	0.4122	0.333	0.3044	0.2431
C	24.087	41.611	38.559	34.934	32.925	30.452
R²	0.9997	0.9806	0.9712	0.965	0.9968	0.9964
Ion leached	Potassium					
	Step 1					
k_t_	0.0685	0.0212	0.0515	0.056	0.0633	0.0671
C	16.148	1.1004	1.9337	2.9437	7.2967	9.1585
R²	0.9780	0.9745	0.9692	0.9864	0.9814	0.9746
	Step 2					
k_t_	0.1023	0.0632	0.1042	0.1148	0.0954	0.0819
C	16.665	1.8335	2.7895	3.9504	7.7943	9.3389
R²	0.9358	0.9944	0.9753	0.9952	0.9918	0.9915
Ion leached	Manganese					
	Step 1					
k_t_		0.0045	0.0124	0.0163	0.0239	0.0407
C		1.4159	3.4269	4.4605	4.9908	5.5832
R²		0.9887	0.9871	0.996	0.9865	0.9955
	Step 2					
k_t_		0.005	0.04	0.0575	0.0785	0.0926
C		1.4224	3.9313	5.2431	6.0011	6.5639
R²		0.9887	0.9954	0.9393	0.981	0.9496

## Data Availability

Not applicable.
